# Author Correction: The thioesterase APT1 is a bidirectional-adjustment redox sensor

**DOI:** 10.1038/s41467-023-40495-4

**Published:** 2023-08-07

**Authors:** Tuo Ji, Lihua Zheng, Jiale Wu, Mei Duan, Qianwen Liu, Peng Liu, Chen Shen, Jinling Liu, Qinyi Ye, Jiangqi Wen, Jiangli Dong, Tao Wang

**Affiliations:** 1 State Key Laboratory of Agrobiotechnology, College of Biological Sciences, China Agricultural University, 100193 Beijing, China; 2 Institute for Agricultural Biosciences, Oklahoma State University, Ardmore, OK 73401 USA; 3 Department of Plant and Soil Sciences, Oklahoma State University, Stillwater, OK 74078 USA

**Keywords:** Stress signalling, Plant signalling, Plant stress responses

Correction to: *Nature Communications* 10.1038/s41467-023-38464-y, published online 17 May 2023

The original version of this Article contained an error in Fig. 5, in which 5e and 5h of this article were inadvertently created using the line charts for 5c and 5d, respectively. This has been corrected in both the PDF and HTML versions of the Article.

