# Supplementary material for: The thioesterase APT1 is a bidirectional-adjustment redox sensor
Source: Nat Commun. 2023 May 17;14:2807. doi: 10.1038/s41467-023-38464-y (PMC10192129; doi:10.1038/s41467-023-38464-y)
Supplement: Supplementary file 1 — Supplementary Information [file 41467_2023_38464_MOESM1_ESM.pdf]

Title: Thioesterase APT1 is a bidirectional adjustment redox sensor

Supplementary Fig. 1

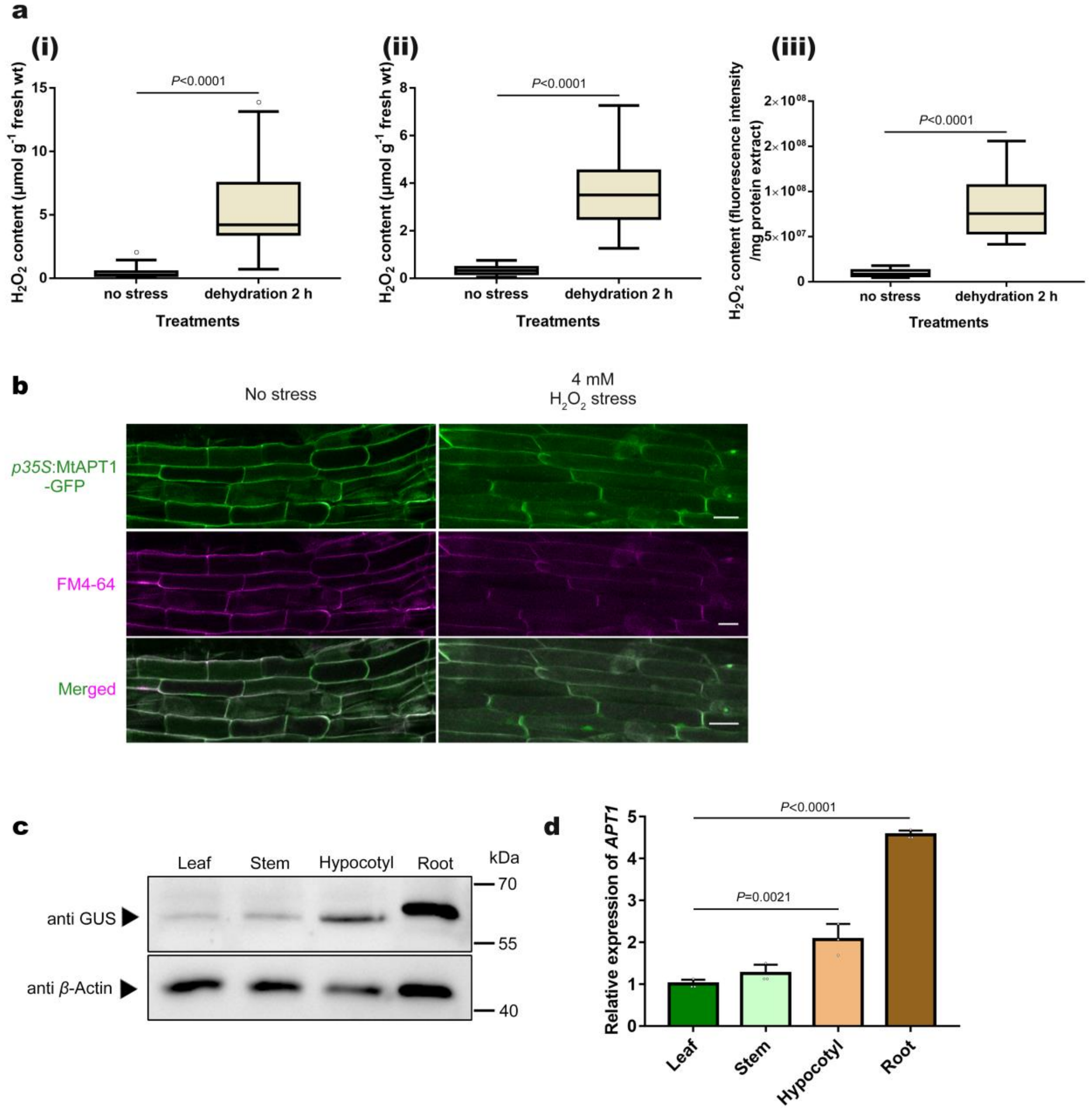

a Differences in the H_2O_2 content in 2-week-old seedling roots of wild-type *Medicago* R108 before and after drought (dehydration) stress for 2 h simulated by 50% PEG-8000, detected by using titanium sulfate (i), trichloroacetic acid (ii) and CM-H_2DCFDA (iii) (i, ii, n=30; iii, n=29). *P* values were two-sided Student's t test. Center lines show the medians; box limits indicate the 25th and 75th percentiles as determined by R software; whiskers extend 1.5 times the interquartile range from the 25th and 75th percentiles, outliers are represented by dots. Box plot statistics: 1a, no stress, upper whisker 0.81, 3rd quartile 0.50, median 0.25, 1st quartile 0.20, lower whisker 0.16; dehydration 2 h, upper whisker 12.68, 3rd quartile 7.29, median 4.21, 1st quartile 3.50, lower whisker 0.72.1b, no stress, upper whisker 0.76, 3rd quartile 0.45, median 0.32, 1st quartile 0.20, lower whisker 0.05; dehydration 2 h, upper whisker 7.27, 3rd quartile 4.44, median 3.51, 1st quartile 2.54, lower whisker 1.26.1c, no stress, upper whisker 17944522, 3rd quartile 11605861, median 8429642, 1st quartile 6675196, lower whisker 4562714; dehydration 2 h, upper whisker 155675248, 3rd quartile 99273325, median 75504830, 1st quartile 55333584, lower whisker 41466035. Source data are provided as a Source

Data file.

b Confocal images of transgenic *M. truncatula* roots expressing APT1-GFP fusion proteins driven by the constitutive CaMV 35S promoter in the *apt1* mutant under normal or 4 mM H_2O_2 stress conditions for 30 min. FM4-64 was used to label the cytoplasmic membrane and excited at a wavelength of 546 nm. Bars = 20 μm.

c Detection of tissue-specific expression by SDS–PAGE and Western blotting analyses of various tissues of transgenic *M. truncatula* expressing GUS proteins driven by the constitutive APT1 promoter (2.0 kb).

d Relative expression of the *MtAPT1* gene in different tissues of the WT. The mean values and SEs were calculated from three independent replicates. *P* values were two-sided nonparametric one-way ANOVA and Tukey's multiple range test. Other lines were no significance with WT.

The source data are provided as a Source Data file.

Supplementary Fig. 2

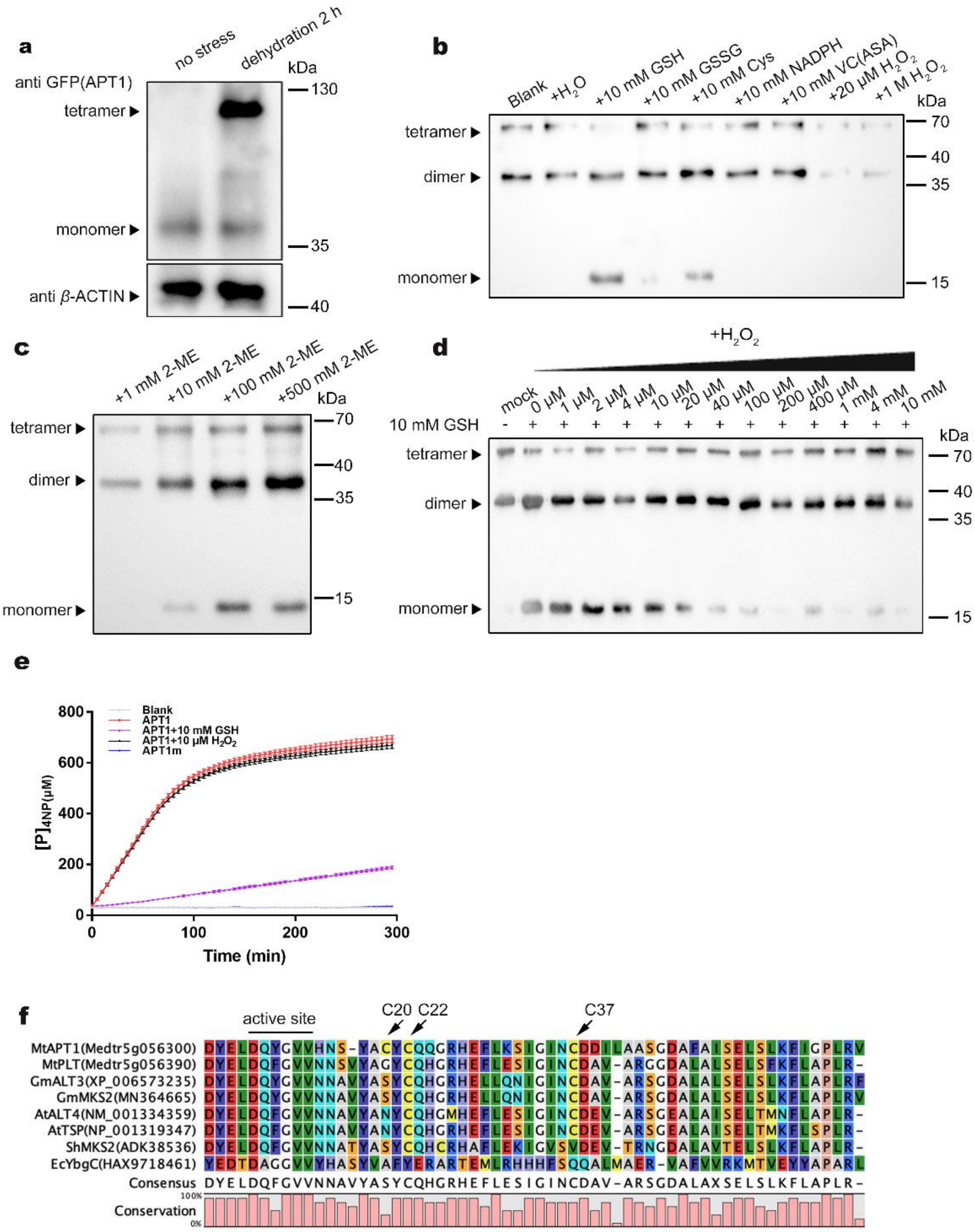

a Western blotting of transgenic *M. truncatula apt1* roots expressing APT1-GFP fusion protein separated on nonreducing SDS–PAGE gels showed APT1 multimerization under no stress and dehydration treatment simulated with 50% PEG-8000.

b Western blotting of the 6×His-APT1 fusion protein separated on nonreducing SDS–PAGE gels after purification (from *E. coli.*) treated with different oxidizing and reducing agents.

c Western blotting of the 6×His-APT1 fusion protein separated on nonreducing SDS–PAGE gels after purification by using different concentrations of 2-ME.

d Western blotting of the 6×His-APT1 fusion protein separated on nonreducing SDS–PAGE gels after different treatments. APT1 (same amount of protein) appeared as monomers after treatment with 10 mM GSH, but further treatment with H_2O_2 (0 μM, 1

μM, 2 μM, 4 μM, 10 μM, 20 μM, 40 μM, 100 μM, 200 μM, 400 μM, 1 mM, 4 mM and 10 mM) promoted APT1 remultimerization at different levels.

e Enzymatic reaction progress curve for APT1, APT1+10 mM GSH, APT1+10 μM H_2O_2, and APT1m (enzyme with a mutation in the active site, DXXGXV mutated to AXXAXA) within 300 min. The results are expressed as the concentration of the product 4-nitrophenolate. The mean values and SEs were calculated from six independent replicates (protein samples with GSH or H_2O_2 or blank).

f Amino acid sequence analysis of MtAPT1 compared with highly homologous genes from other species, including *Glycine max*, *Arabidopsis thaliana*, *Solanum habrochaites* and *Escherichia coli*.

The source data are provided as a Source Data file.

Supplementary Fig. 3

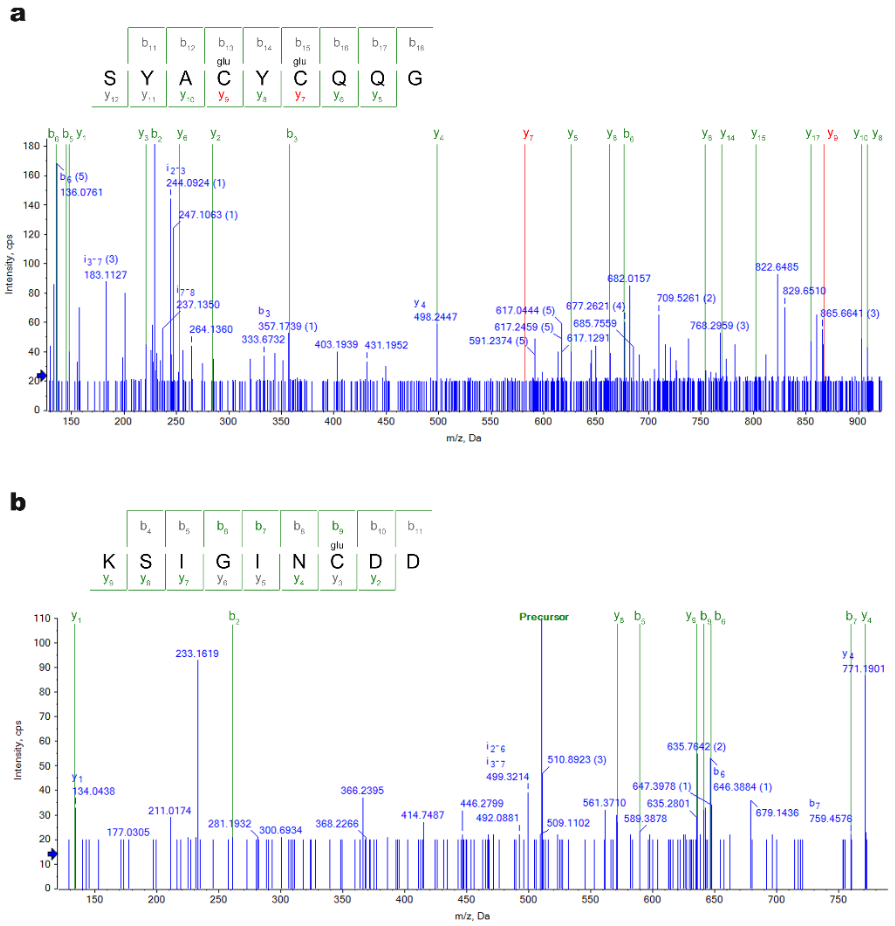

LC–MS/MS spectra of the modification of APT1 with 10 mM GSH. The C20 and C22 peptides are shown in **a**, and the C37 peptide is shown in **b**. Green indicates the detected amino acid ion fragments, grey/red indicates no detected amino acid ion fragments, and glu refers to cysteine with S-glutathionylation.

Supplementary Fig. 4

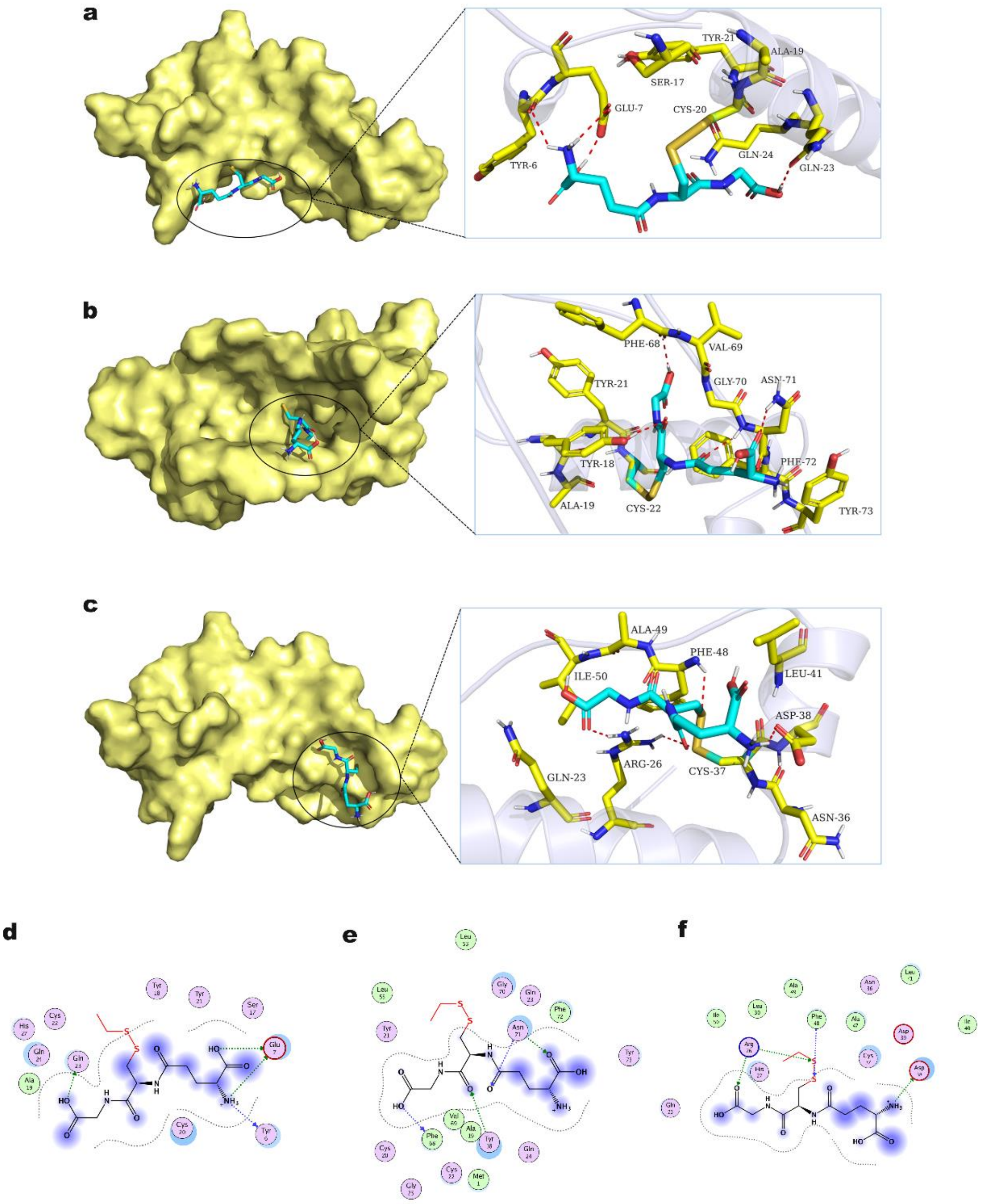

Binding model of GSH on the molecular surface of C20, C22, and C37 of APT1 in three dimensions determined by molecular docking. GSH is coloured cyan, and the molecular surfaces of C20 (**a**), C22 (**b**), and C37 (**c**) of APT1 are coloured pale yellow. In the magnified view, GSH is coloured cyan, the surrounding residues in the binding pockets are coloured yellow, and the backbone of the receptor is depicted as a white cartoon with transparency. **d**, **e**, and **f** show the two-dimensional models of **a**, **b**, and **c**, respectively.

Supplementary Fig. 5

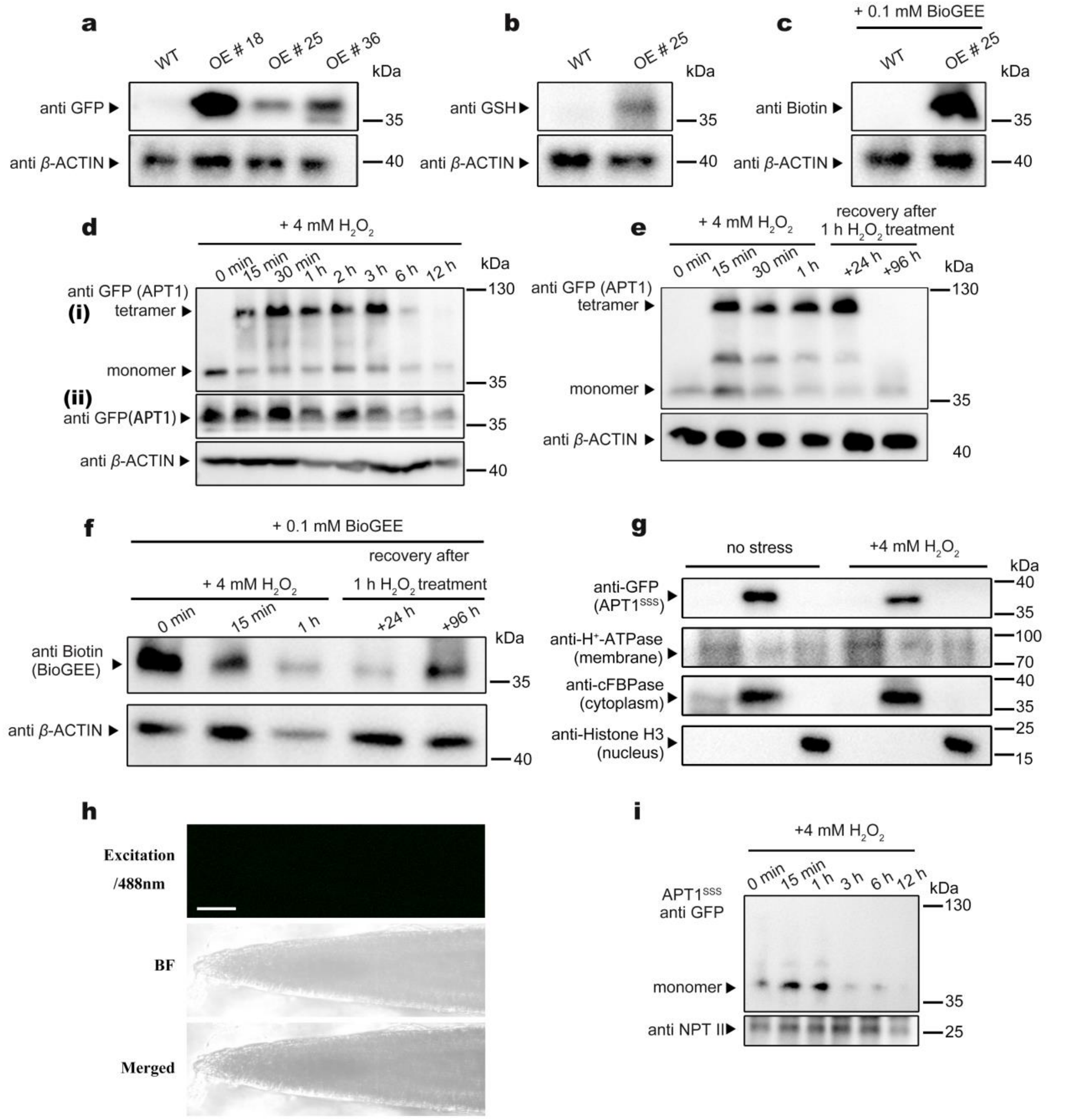

Immunoblotting of the *APT-GFP-overexpressing* stable transgenic lines #18, #25 and #36 with GFP antibody (**a**), GSH antibody (**b**), and biotin antibody (**c**).

d Western blot of proteins separated on nonreducing SDS–PAGE gels showed APT1-GFP fusion protein multimerization and demultimerization in transgenic *M. truncatula apt1* roots exposed to 4 mM H_2O_2; detection was performed with anti-GFP (APT1) (i) antibodies. Western blot of proteins separated on reducing SDS–PAGE gels showed APT1-GFP fusion protein degradation in transgenic *M. truncatula apt1* roots when exposed to 4 mM H_2O_2; detection was performed with anti-GFP (APT1) and anti-*β*-actin (control protein) (ii) antibodies. A i to A ii show results from the same sample with the same loading volume (10 μl).

e Western blot of proteins separated on nonreducing SDS–PAGE gels showed APT1-GFP fusion protein multimerization and demultimerization in transgenic *M. truncatula apt1* roots exposed to 4 mM H_2O_2 and after recovery (1 h of 4 mM H_2O_2 stress followed by 24 h of nonstressed conditions in normal hydroponic solution); detection was

performed with anti-GFP (APT1) and anti-*β*-actin (control protein) antibodies, and the results showed the same sample with the same loading volume (10 μl).

f Western blot of proteins separated on nonreducing SDS–PAGE gels showed APT1-GFP fusion protein multimerization, demultimerization and S-glutathionylation in transgenic *M. truncatula apt1* roots when exposed to 4 mM H_2O_2 and after recovery; detection was performed with anti-GFP (APT1), antibiotin and anti-*β*-actin (control protein) antibodies. Biotinylated glutathione ethyl ester (BioGEE) is a cell-permeable biotinylated glutathione analogue used for the detection of S-glutathionylation reactions. Before oxidative stress, the cells were treated with 0.1 mM BioGEE for 1 h for the S-glutathionylation of labelled proteins.

g Western blot of separation of transgenic *M. truncatula* roots expressing $APT1^{SSS}$-GFP fusion proteins driven by the constitutive CaMV 35S promoter in the *apt1* mutant in the nonstressed state or after 4 mM H_2O_2 treatment for 30 min.

h Confocal images of the WT root tip with excitation at 488 nm and emission at 525 nm using the same scan parameter settings as in Fig. 4A, which was used as a negative control. Bars = 20 μm.

i Western blot of proteins separated on nonreducing SDS–PAGE gels showed that the $APT1^{SSS}$-GFP (C20SC22SC37S) fusion protein did not multimerize in transgenic *M. truncatula apt1* roots after 4 mM H_2O_2 treatment; detection was performed using anti-GFP (APT1), anti-*β*-actin (control protein) and anti-NPTII (kanamycin resistance, control protein of transient transgene expression) antibodies.

The source data are provided as a Source Data file.

Supplementary Fig. 6

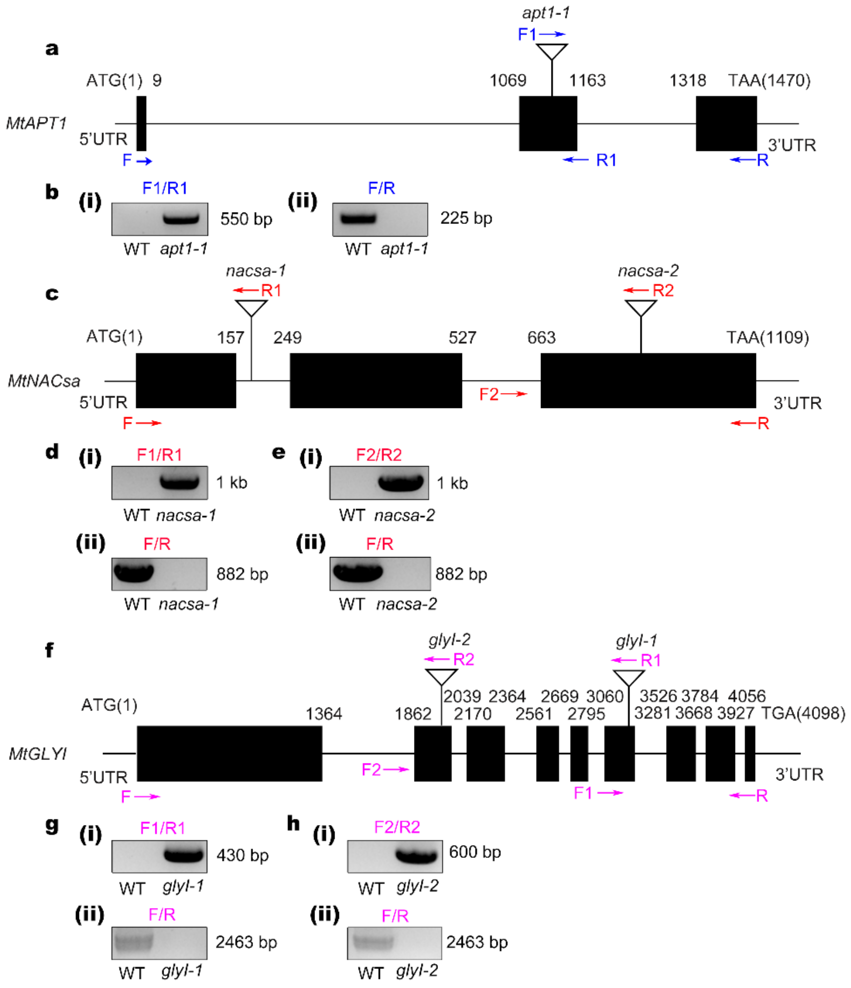

Molecular identification of *apt1*, *nacsa*, and *glyI* mutants. *MtAPT1* (**a**), *MtNACsa* (**c**), and *MtGLYI* (**f**) gene structure and *Tnt1* insertion sites. Exons are indicated by solid boxes, and introns are indicated by boldface lines. *Tnt1* is indicated by triangles. PCR identification of the *Tnt1* insertion site and homozygous lines of the *apt1-1* (**b**-i), *nacsa-1* (**d**-i), *nacsa-2* (**e**-i), *glyI-1* (**g**-i), and *glyI-2* (**h**-i) mutants detected from DNA samples. PCR identification of the *Tnt1* insertion site and homozygous lines of the *apt1-1* (**b** ii), *nacsa-1* (**d**-ii), *nacsa-2* (**e**-ii), *glyI-1* (**g**-ii), *and glyI-2* (**h**-ii) mutants detected from cDNA samples. The primers used for detection are shown in different colours.

Supplementary Fig. 7

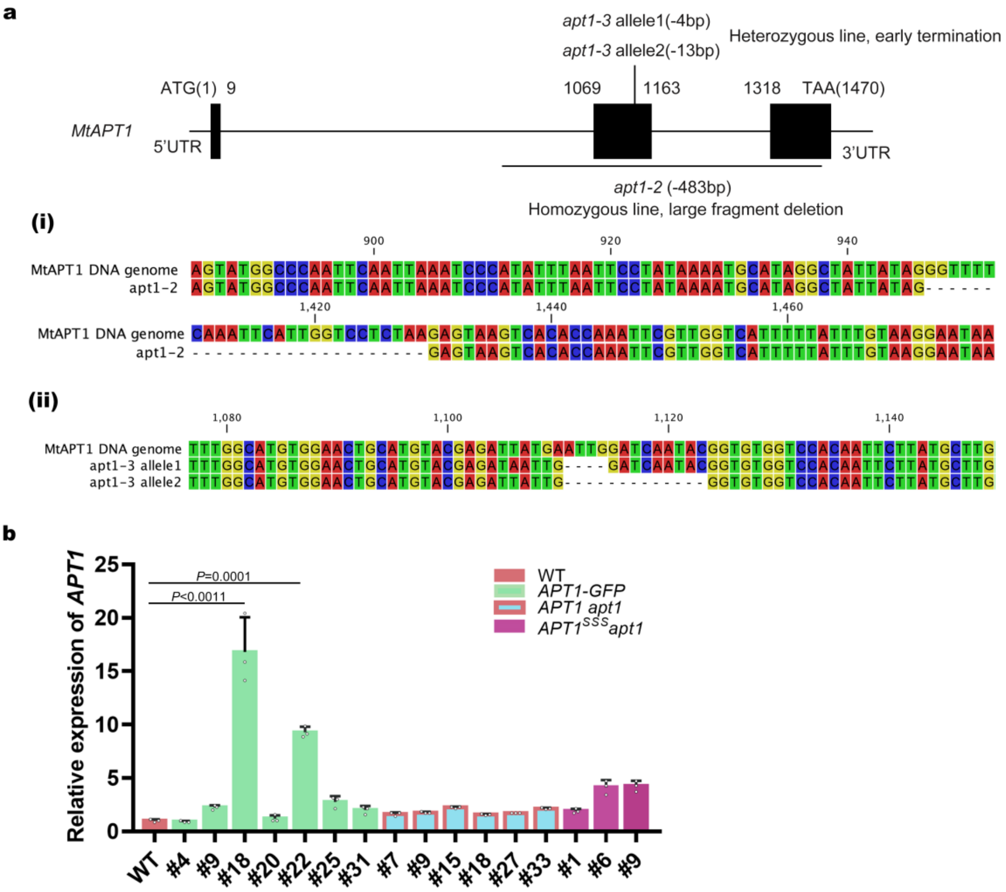

Molecular identification of *apt1-2* and *apt1-3* mutants and transgenic lines of *APT1* and *APT1*-specific tissue expression.

a *MtAPT1* gene structure and the base-site-deletion mutants *apt1-2* (i) and *apt1-3* (ii).

b Relative *MtAPT1* expression in the WT and different transgenic lines. The mean values and SEs were calculated from three technical replicates. The *p35S:APT1-GFP* stable line #25 was used to detect multimers of APT1 after oxidative stress. The *pAPT1:APT1-3×flag/apt1* stable line #7 and the *pAPT1:APT1*^{SSS}*-3×flag/apt1* stable line #1 were used to detect *GLYI* expression and determine the GSH/GSSG ratio, ROS (H_2O_2)/GSH labelling and NBT/DAB staining. The mean values and SEs were calculated from three independent replicates. *P* values were two-sided nonparametric one-way ANOVA and Tukey's multiple range test. Other lines were no significance with WT.

The source data are provided as a Source Data file.

Supplementary Fig. 8

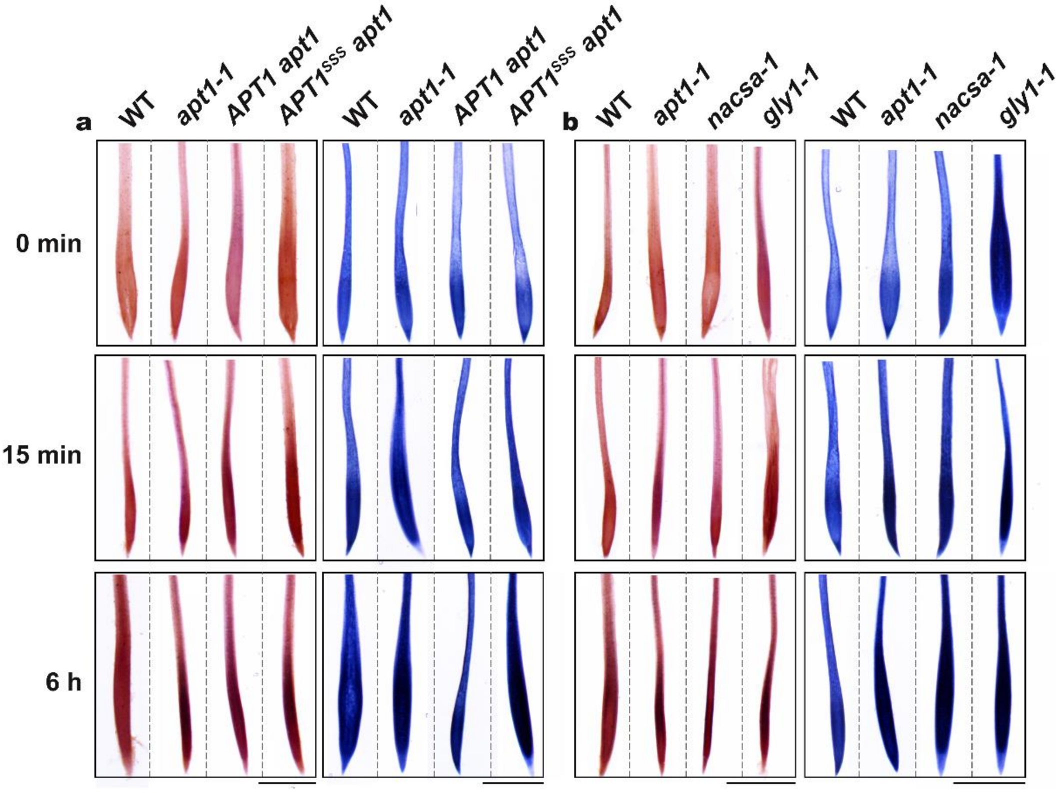

Multimerized APT1 transports signals through NACsa to reduce ROS accumulation. DAB (brown) and NBT (blue) staining of WT, *apt1-1* mutant, *pAPT1:APT1-3×flag/apt1* and *pAPT1:APT1*^{SSS}*-3×flag/apt1* (**a**) and *apt1*, *nacsa*, *glyI* (b) after 0 h, 15 min, and 6 h of 4 mM H_2O_2 stress. Bars = 1 mm.

Supplementary Table 1. Primers used in this study.

For real-time PCR		
Primer name		Primer sequences (5'-3')
MtAPT1	Forward primer	GTCGTCATGAATTTTTGAAAA
	Reverse primer	CTTAGAGGACCAATGAATTTG
MtGLYI	Forward primer	ATATTGTAAAGGCGAAAGGGGGAA
	Reverse primer	CTTTTTCATAGAAGGCTATGACAC
For cloning and genotyping		
pCAMBIA1307-*MtAPT1*-3×FLAG	Forward primer	TGTCGACGGATCCGCGAGCTCATGGCACCAGTCGATTATGAA
	Reverse primer	ATGGTCTTTGTAGTCACTAGTCAAATAAAAATTACCAACGAAT
pCAMBIA1307-MtAPT1-GFP	Forward primer	TGTCGACGGATCCGCGAGCTCATGGCACCAGTCGATTATGAA
	Reverse primer	AGTCAGATCTACCATTCTAGACAAATAAAAATTACCAACGAAT
pET30a(+)-*MtAPT1*	Forward primer	GGAATTCATGGCACCAGTCGATTATG
	Reverse primer	CCCAAGCTTTTACAAATAAAAATTACCAACGAAT
pE3025-*MtAPT1*-GFP	Forward primer	TCGAGCTCAAGCTTCGAATTCATGGCACCAGTCGATTATGAA
	Reverse primer	GGATCCCGGGCCCGCGGTACCGCAAATAAAAATTACCAACGAAT
pCAMBIA1381-pAPT1:GUS	Forward primer	TGGGCCCGGCGCGCCGAATTCAGATTGGGTTTACAATTAAAAT
	Reverse primer	CCATGGTGGACTCCTCTTAAAGCTTCTTTTATGATCATTCTGAACCC
pCAMBIA1381-pAPT1:*MtAPT1*-3×FLAG	Forward primer	GAATGATCATAAAAGAAGCTTATGGCACCAGTCGATTAT
	Reverse primer	GGTGGACTCCTCTTAAAGCTTCTACTTATCGTCATCGTC
pCAMBIA1381-pAPT1:*MtAPT1*-GFP	Forward primer	GAATGATCATAAAAGAAGCTTATGGCACCAGTCGATTAT
	Reverse primer	GGTGGACTCCTCTTAAAGCTTTTACTTGTACAGCTCGTCCAT
MtAPT1-C20S (site mutant)	Forward primer	TCTTATGCTTCTTATTGCCAAC
	Reverse primer	GTTGGCAATAAGAAGCATAAGA
MtAPT1-C22S	Forward primer	TCTTATGCTTGTTATTCCCAAC
	Reverse primer	GTTGGGAATAACAAGCATAAGA
MtAPT1-C37S	Forward primer	GGTATCAATTCTGATGATATC
	Reverse primer	GATATCATCAGAATTGATACC

MtAPT1-C20SC22S	Forward primer	TCTTATGCTTCTTATTCCCAAC
	Reverse primer	GTTGGGAATAAGAAGCATAAGA
NF10049 (*Tnt1* insertion site)	Forward primer	ATCTAGTGGAGAAGCAGAGACAGTAG
	Reverse primer	AACCACGACCCCGAGAGGAGCAACT
NF5250 (*Tnt1* insertion site)	Forward primer	ATGCAAGGTGAATTAGAATTACCAC
	Reverse primer	CCAAACACATTGATGTGAGATATCAT
NF9803 (*Tnt1* insertion site)	Forward primer	GCAATTTGCAGCTTGATGATTGGGTG
	Reverse primer	CCTCAGCTTTCATGGTATCAGGCTT
NF15130 (*Tnt1* insertion site)	Forward primer	ACAAAGTTGGCTACCAATCCAAC
	Reverse primer	AATTCTTATGCTTGTTATTGCCA
NF20885 (*Tnt1* insertion site)	Forward primer	AATCACAACAGATGAGATTTGGATAT
	Reverse primer	AACCACGACCCCGAGAGGAGCAACT
DT1-BsF-*MtAPT1* (for CRISPR)	Forward primer	ATATATGGTCTCGATTGGAATTGGATCAATACGGTGGTT
DT2-BsR-*MtAPT1*	Reverse primer	ATTATTGGTCTCGAAACATTCATAATCTCGTACATGC
DT1-F0-*MtAPT1*	Forward primer	TGGAATTGGATCAATACGGTGGTTTTAGAGCTAGAAATAGC
DT2-R0-*MtAPT1*	Reverse primer	AACATTCATAATCTCGTACATGCAAGCCCTGCTGTTCGTCTAG

149

Supplementary Table 2. The significance of the mean value of the ROS detection fluorescence intensity for each point of Fig. 5 **c** and **d**.

no stress														
	**1**	**2**	**3**	**4**	**5**	**6**	**7**	**8**	**9**	**10**	**11**	**12**	**13**	**14**
**WT**	b	a	b	c	c	c	bc	a	a	a	b	a	a	a
apt1	b	a	b	bc	c	bc	c	a	a	a	ab	a	a	a
**APT1/*apt1***	b	a	b	c	bc	bc	bc	a	a	a	ab	a	a	a
**APT1^{SSS}/*apt1***	b	a	b	bc	bc	bc	ab	a	a	a	a	a	a	a
nacsa	b	a	b	b	b	b	bc	a	a	a	ab	a	a	a
glyI	a	a	a	a	a	a	a	a	a	a	a	a	a	a

oxidative stress 15 min														
	**1**	**2**	**3**	**4**	**5**	**6**	**7**	**8**	**9**	**10**	**11**	**12**	**13**	**14**
**WT**	b	b	c	c	b	b	b	b	b	a	b	b	a	b
apt1	ab	a	a	ab	a	ab	a	a	ab	a	ab	ab	a	ab
**APT1/*apt1***	ab	b	bc	c	b	b	b	b	b	a	b	ab	a	b
**APT1^{SSS}/*apt1***	ab	ab	ab	bc	ab	ab	a	a	ab	a	ab	ab	a	ab
nacsa	a	ab	ab	bc	ab	a	a	a	ab	a	b	ab	a	ab
glyI	b	ab	a	a	a	a	a	a	a	a	a	a	a	a

The different words indicate a significant difference in different lines (*P*<0.05), which performed by two-sided nonparametric one-way ANOVA and Tukey's multiple range test.

Supplementary Table 3. The significance of the mean value of the GSH detection fluorescence intensity for each point of Fig 5 **e**, **h**, and **k**.

no stress														
	**1**	**2**	**3**	**4**	**5**	**6**	**7**	**8**	**9**	**10**	**11**	**12**	**13**	**14**
**WT**	**a**	**a**	**a**	**ab**	**a**	**a**	**a**	**a**	**a**	**a**	**a**	**a**	**a**	**a**
apt1	**a**	**a**	**a**	**ab**	**b**	**a**	**ab**	**ab**	**bc**	**b**	**ab**	**ab**	**a**	**ab**
**APT1/*apt1***	**a**	**ab**	**a**	**a**	**a**	**a**	**a**	**a**	**b**	**bc**	**bc**	**ab**	**b**	**c**
**APT1^{SSS}/*apt1***	**a**	**ab**	**a**	**ab**	**a**	**a**	**a**	**a**	**ab**	**b**	**bc**	**b**	**b**	**c**
nacsa	**a**	**a**	**a**	**a**	**a**	**a**	**a**	**a**	**bc**	**b**	**abc**	**ab**	**a**	**bc**
glyI	**b**	**b**	**b**	**b**	**b**	**b**	**b**	**b**	**c**	**c**	**c**	**c**	**b**	**d**

oxidative stress 15 min														
	**1**	**2**	**3**	**4**	**5**	**6**	**7**	**8**	**9**	**10**	**11**	**12**	**13**	**14**
**WT**	**a**	**a**	**a**	**a**	**a**	**a**	**a**	**a**	**b**	**a**	**a**	**a**	**a**	**a**
apt1	**b**	**b**	**b**	**b**	**bc**	**bc**	**b**	**b**	**c**	**b**	**bc**	**cd**	**bc**	**bc**
**APT1/*apt1***	**a**	**a**	**a**	**a**	**a**	**a**	**a**	**a**	**a**	**a**	**a**	**b**	**a**	**a**
**APT1^{SSS}/*apt1***	**b**	**b**	**b**	**b**	**bc**	**bc**	**b**	**bc**	**c**	**b**	**b**	**c**	**b**	**b**
nacsa	**b**	**b**	**b**	**b**	**b**	**b**	**b**	**c**	**c**	**b**	**bc**	**c**	**c**	**c**
glyI	**c**	**c**	**c**	**c**	**c**	**c**	**b**	**c**	**c**	**b**	**c**	**d**	**c**	**c**

recovery 24 h after oxidative stress 15 min														
	**1**	**2**	**3**	**4**	**5**	**6**	**7**	**8**	**9**	**10**	**11**	**12**	**13**	**14**
**WT**	**a**	**a**	**a**	**a**	**a**	**a**	**a**	**a**	**b**	**ab**	**ab**	**ab**	**ab**	**a**
apt1	**b**	**bc**	**b**	**b**	**b**	**c**	**b**	**c**	**cd**	**c**	**c**	**c**	**b**	**a**
**APT1/*apt1***	**a**	**a**	**a**	**a**	**a**	**ab**	**a**	**a**	**a**	**a**	**a**	**a**	**a**	**a**
**APT1^{SSS}/*apt1***	**b**	**c**	**b**	**b**	**b**	**c**	**b**	**c**	**cd**	**bc**	**bc**	**bc**	**ab**	**a**
nacsa	**b**	**b**	**b**	**b**	**b**	**bc**	**a**	**b**	**bc**	**ab**	**abc**	**ab**	**ab**	**a**
glyI	**b**	**bc**	**b**	**b**	**b**	**c**	**b**	**c**	**d**	**bc**	**c**	**bc**	**ab**	**a**

The different words indicate a significant difference in different lines ($P<0.05$), which performed by two-sided nonparametric one-way ANOVA and Tukey's multiple range test.

Supplementary Methods

Determination of the H_2O_2 level by trichloroacetic acid (TCA)

One hundred milligrams of frozen plant tissue was homogenized with the addition of 1 mL of 0.1% (w/v) TCA, which was kept on ice at all times. Then, the sample was mixed well by vortexing and centrifuged at 15,000×g for 15 min at 4°C. Then, 2 mL tubes were prepared to perform the reaction by adding 0.5 mL of the supernatant and 0.5 mL of 10 mM phosphate buffer (pH 7.0). For the Blank, 0.5 mL of 0.1% (w/v) TCA was used instead of the supernatant. Then, 1 mL of potassium iodide was added to initiate the reaction as quickly as possible. The sample was mixed gently and left in the dark for 10 min. Then, 300 μL of the reaction mixture was transferred to a 96-well plate, and the absorbance at 390 nm was measured in a Multimode microplate reader (Spark®, Tecan, Switzerland). The concentration and content of hydrogen peroxide in plant tissue were calculated using a standard curve[1].

Determination of the H_2O_2 level by CM-H_2DCFDA

Control and treated tissues were harvested, and nearly 100 mg of tissue was ground in liquid nitrogen. The ground tissue powder was placed in a preweighed 2 mL tube with 1 mL of 10 mM Tris-HCl (pH 7.2). The sample was centrifuged at 12,000×g for 20 min at 4°C. Then, the supernatant was transferred to a fresh 2 mL tube. The instrument was blanked with 10 mM Tris-HCl (pH 7.2). One hundred microlitres of supernatant was diluted with 900 μL of 10 mM Tris-HCl (pH 7.2). Then, 10 μL of 1 mM CM-H_2DCFDA (final concentration will be 10 μM) (C6827, Thermo) was added to the first sample, and after 1 min, 10 μL of CM-H_2DCFDA was added to the next sample. The rest of the samples were vortexed and incubated in the dark for 10 min. The fluorescence values of samples treated with CM-H_2DCFDA were measured by a fluorometer (RF-5301 PC, Shimadzu, Japan). The control was set by adding 100 μL of plant extract + 900 μL of Tris-HCl (pH 7.2), inverting and mixing (using parafilm), and the values were read in a fluorometer. This background fluorescence value was deducted from all readings. The protein concentration in all samples was estimated using Bradford reagent (P0010, Beyotime, China), and ROS levels are expressed as relative fluorescence units/mg of protein extract. In this experiment, to rule out doubts about CM-H_2DCFDA not being completely specific to ROS, we performed measurements on equal aliquots with catalase (300 U/mL) added to one sample and repeated the measurement 6 times, subtracting the catalase-insensitive background from the experimental value[2].

Supplementary references

1. Antoniou, C., Savvides, A., Georgiadou, E.C., Fotopoulos, V. (2018). Spectrophotometric quantification of reactive oxygen, nitrogen and sulfur species in plant samples. In: Alcázar, R., Tiburcio, A. (eds) Polyamines. Methods in Molecular Biology, vol 1694. Humana Press, New York, NY. https://doi.org/10.1007/978-1-4939-7398-9_16.
2. Jambunathan, N. (2010). Determination and detection of reactive oxygen species (ROS), lipid peroxidation, and electrolyte leakage in plants. In: Sunkar, R. (eds)

Plant Stress Tolerance. Methods in Molecular Biology, vol 639. Humana Press. https://doi.org/10.1007/978-1-60761-702-0_18.
