# Supplementary material for: The thioesterase APT1 is a bidirectional-adjustment redox sensor
Source: Nat Commun. 2023 May 17;14:2807. doi: 10.1038/s41467-023-38464-y (PMC10192129; doi:10.1038/s41467-023-38464-y)
Supplement: Supplementary file 3 — Reporting Summary [file 41467_2023_38464_MOESM3_ESM.pdf]

nature portfolio

Corresponding author(s): Tao Wang
Last updated by author(s): Apr 7, 2023

Reporting Summary

Nature Portfolio wishes to improve the reproducibility of the work that we publish. This form provides structure for consistency and transparency in reporting. For further information on Nature Portfolio policies, see our Editorial Policies and the Editorial Policy Checklist.

Statistics

For all statistical analyses, confirm that the following items are present in the figure legend, table legend, main text, or Methods section.

n/a	Confirmed	
☐	☒	The exact sample size (*n*) for each experimental group/condition, given as a discrete number and unit of measurement
☐	☒	A statement on whether measurements were taken from distinct samples or whether the same sample was measured repeatedly
☐	☒	The statistical test(s) used AND whether they are one- or two-sided<br>*Only common tests should be described solely by name; describe more complex techniques in the Methods section.*
☐	☒	A description of all covariates tested
☒	☐	A description of any assumptions or corrections, such as tests of normality and adjustment for multiple comparisons
☐	☒	A full description of the statistical parameters including central tendency (e.g. means) or other basic estimates (e.g. regression coefficient) AND variation (e.g. standard deviation) or associated estimates of uncertainty (e.g. confidence intervals)
☐	☒	For null hypothesis testing, the test statistic (e.g. *F*, *t*, *r*) with confidence intervals, effect sizes, degrees of freedom and *P* value noted<br>*Give P values as exact values whenever suitable.*
☒	☐	For Bayesian analysis, information on the choice of priors and Markov chain Monte Carlo settings
☒	☐	For hierarchical and complex designs, identification of the appropriate level for tests and full reporting of outcomes
☒	☐	Estimates of effect sizes (e.g. Cohen's *d*, Pearson's *r*), indicating how they were calculated

Our web collection on statistics for biologists contains articles on many of the points above.

Software and code

Policy information about availability of computer code

Data collection	ImageJ software 1.52a<br>NIS-Elements Viewer software 4.20<br>MOE software v2018.0172<br>PeakView software v2.2<br>MO. Control software<br>geNorm software<br>CFX Manager™ Software<br>Compass software v6.2
Data analysis	SPSS software 22<br>GraphPad Prism software 7<br>MO.Affinity Analysis software 2.3

For manuscripts utilizing custom algorithms or software that are central to the research but not yet described in published literature, software must be made available to editors and reviewers. We strongly encourage code deposition in a community repository (e.g. GitHub). See the Nature Portfolio guidelines for submitting code & software for further information.

Data

Policy information about availability of data

All manuscripts must include a data availability statement. This statement should provide the following information, where applicable:

- Accession codes, unique identifiers, or web links for publicly available datasets
- A description of any restrictions on data availability
- For clinical datasets or third party data, please ensure that the statement adheres to our policy

All data used in this study is freely available as a supplement to this manuscript (Source Data).

Human research participants

Policy information about studies involving human research participants and Sex and Gender in Research.

Reporting on sex and gender	Not Applicable
Population characteristics	Not Applicable
Recruitment	Not Applicable
Ethics oversight	Not Applicable

Note that full information on the approval of the study protocol must also be provided in the manuscript.

Field-specific reporting

Please select the one below that is the best fit for your research. If you are not sure, read the appropriate sections before making your selection.

☒ Life sciences ☐ Behavioural & social sciences ☐ Ecological, evolutionary & environmental sciences

For a reference copy of the document with all sections, see nature.com/documents/nr-reporting-summary-flat.pdf

Life sciences study design

All studies must disclose on these points even when the disclosure is negative.

Sample size	Sample sizes were selected that are widely used in the field, for example see Wu et al. Nature, 578, pages 577–581 (2020). No statistical tests were used to predetermine sample size.
Data exclusions	No results or data were excluded from this study.
Replication	All replicates were successful, and nothing has been excluded from this study. Values of counting number (n) and numbers of biological replicates are indicated in each figure legends.
Randomization	Plants were grown in carefully randomized growth in specific conditions which described in methods and materials, and pots changed randomly to ensure all plants received identical conditions. Seedlings were grown on sterile MS-agar medium culture plates. They were randomly mixed and processed.
Blinding	Investigators setup and performed experiments, this, researchers did not fully forget the experimental setup while collecting data.

Reporting for specific materials, systems and methods

We require information from authors about some types of materials, experimental systems and methods used in many studies. Here, indicate whether each material, system or method listed is relevant to your study. If you are not sure if a list item applies to your research, read the appropriate section before selecting a response.

Materials & experimental systems

n/a	Involved in the study
☐	☒ Antibodies
☒	☐ Eukaryotic cell lines
☒	☐ Palaeontology and archaeology
☒	☐ Animals and other organisms
☒	☐ Clinical data
☒	☐ Dual use research of concern

Methods

n/a	Involved in the study
☒	☐ ChIP-seq
☒	☐ Flow cytometry
☒	☐ MRI-based neuroimaging

Antibodies

Antibodies used

Anti-GFP (Abmart, M20004S), Anti-GUS (Abmart, P26299-1), Anti-His (Proteintech, 66005-1-Ig), Anti-Kan/NBTII(Abcam, ab60018), Anti-H+-ATPase(Agrisera, AS07260), Anti-cFBPase (Agrisera, AS04043), Anti-histone H3 (Agrisera, AS10710), HRP conjugate antibody (SA10001, Invitrogen), H2O2 fluorescence probe H2DCFDA (Sigma, D6883), GSH fluorescence probe ThiolTracker™ Violet dye (Invitrogen, T10095) , GSH monoclonal antibody (MA1-7620, Invitrogen)

Validation

Validation statement for Anti-GFP (Abmart, M20004S, mouse) can be found at the product website: http://www.ab-mart.com.cn/page.aspx?node=%2060%20&id=%20971
Validation statement for Anti-GUS (Abmart, P26299-1, rabbit) can be found at the product website: http://www.ab-mart.com.cn/page.aspx?node=%2060%20&id=%2049655
Validation statement for Anti-His (Proteintech, 66005-1-Ig, mouse) can be found at the product website: http://www.ptgcn.com/products/His-Tag-Antibody-66005-1-Ig.htm
Validation statement for Anti-Kan/NBTII(Abcam, ab60018, mouse) can be found at the product website: https://www.abcam.cn/kan-antibody-4b4d1-ab60018.html
Validation statement for Anti-H+-ATPase(Agrisera, AS07260, rabbit) can be found at the product website: https://www.agrisera.com/en/artiklar/hatpase-plasma-membrane-hatpase.html
Validation statement for Anti-cFBPase (Agrisera, AS04043, rabbit) can be found at the product website: https://www.agrisera.com/en/artiklar/cfbpase-cytosolic-fructose-16-bisphosphatase-marker-for-cytoplasm.html
Validation statement for Anti-histone H3 (Agrisera, AS10710, mouse) can be found at the product website: https://www.agrisera.com/en/artiklar/h3-histone-h3.html
Validation statement for HRP conjugate antibody (SA10001, Invitrogen, mouse) can be found at the product website: https://www.thermofisher.cn/order/catalog/product/SA10001?SID=srch-hj-SA10001
Validation statement for H2O2 fluorescence probe H2DCFDA (Sigma, D6883) can be found at the product website: https://www.sigmaaldrich.cn/CN/zh/product/sigma/d6883
Validation statement for GSH fluorescence probe ThiolTracker™ Violet dye (Invitrogen, T10095) can be found at the product website: https://www.thermofisher.cn/order/catalog/product/T10095?SID=srch-hj-T10095
Validation statement for GSH monoclonal antibody (MA1-7620, Invitrogen) can be found at the product website: https://www.thermofisher.cn/cn/zh/antibody/product/Glutathione-Antibody-clone-D8-Monoclonal/MA1-7620
